# The Validity of Rapid Malaria Test and Microscopy in Detecting Malaria in a Preelimination Region of Egypt

**DOI:** 10.1155/2016/4048032

**Published:** 2016-03-21

**Authors:** Maysa Mohamed Kamel, Samar Sayed Attia, Gomaa Desoky Emam, Naglaa Abd El Khalek Al Sherbiny

**Affiliations:** ^1^Medical Parasitology Department, Faculty of Medicine, Cairo University, Cairo, Egypt; ^2^Medical Parasitology Department, Faculty of Medicine, Fayoum University, Fayoum, Egypt; ^3^Department of Community Medicine, Faculty of Medicine, Fayoum University, Fayoum, Egypt

## Abstract

*Background*. Malaria is a leading cause of morbidity and mortality worldwide. Rapid and accurate diagnosis of malaria would improve control measures and reduce morbidity and mortality.* Objective*. The aim of this study was to assess the prevalence of malaria in high risk foci in Egypt and the effectiveness of rapid diagnostic tests in diagnosis and subsequently control of malaria.* Methodology*. A total number of 600 cases of both sexes with different ages were included in the present study. Cases were included in 2 groups; first group (500 cases) were randomly selected from households in Fayoum Governorate and second group (100 cases) were admitted to Fayoum Fever Hospital with signs suggestive of malaria. Cases were subjected to detailed history taking, clinical examination, microscopic examination of thin and thick blood films, and immunological test to detect plasmodial antigens.* Results*. A total of 3 positive cases were detected by rapid diagnostic tests (RDTs). Out of these 3 cases, one case was positive for malaria parasite by microscopic examination of blood films. All positive cases in the study had history of travel to malaria endemic areas.* Conclusion*. RDTs are simple and effective for rapid diagnosis of malaria to help in implication of control measures in different localities.

## 1. Introduction 

Malaria is an infectious disease caused by parasites belonging to the genus* Plasmodium* which affects about 3.4 billion people [1], of which 2.57 billion are at risk for* P. falciparum* and 2.5 billion are at risk for* P. vivax*. Meanwhile,* P. malariae* and* P. ovale* contribute a very small proportion of malaria infections [2].

According to WHO, malaria caused 1.2 million deaths worldwide [3]. In Africa, malaria is recognized as the 2nd leading cause of death from infectious diseases after HIV/AIDS [4].

Malaria is a largely preventable and treatable disease and WHO defines malaria control as “reducing the disease burden to a level at which it is no longer a public health problem” [5]. There is a wide range of malaria control interventions including ITNs [6], indoor residual spraying (IRS) [7], the use of artemisinin combination therapy (ACT) as first-line therapy [8], and improved diagnosis using rapid diagnostic tests (RDTs) [9, 10].

The overlapping of malaria symptoms with other tropical diseases impairs diagnostic specificity, which can promote the indiscriminate use of antimalarials and compromise the quality of care for patients with nonmalarial fevers in endemic areas [11–13]. The accuracy of malaria diagnosis can be greatly enhanced by combining clinical- and parasite-based findings [14].

The standard method of malaria diagnosis is by the microscopic examination of Giemsa-stained blood smears [15]. Expert microscopy gives information about parasite stage and parasitaemia. However, maintaining a high standard of microscopy requires experienced technicians, a continuous supply of good quality staining reagents, and properly maintained microscopes.

Rapid diagnostic tests (RDTs) that detect malaria parasite proteins by immunochromatography have been used as complementary detection method for malaria diagnosis [16].

RDTs detect a variety of proteins, including* P. falciparum* histidine-rich protein 2 (PfHRP2) and* P. falciparum* lactate dehydrogenase (PfLDH), both specific to* P. falciparum*, and also* Plasmodium* LDH (pLDH) and aldolase, enzymes shared by the 5 human pathogenic* Plasmodium* species [17].

Malaria has been a well-known disease in Egypt since ancient times [18]. Before the eradication of malaria from Egypt, high levels of the infection appeared to have been limited to certain parts of the country and to be strictly linked to its geology [19]. The last focus for malaria was in Fayoum which became free from transmission of malaria since 1998 and Egypt was certificated as free of malaria due to strong national control program applied by Ministry of Health in cooperation with WHO. According to WHO [3], Egypt and other countries have interrupted transmission and are in the prevention of reintroduction phase of malaria control.

As malaria control improves, surveillance will become necessary to identify persistent and hot spots of infection as well as localized areas where control measures are not effective so as to apply such control measures where infection is diagnosed. This can be achieved by the use of laboratory diagnostic methods [20] to support clinical data in estimating burden of malaria.

## 2. Patients and Methods

The present study was conducted on a total of 600 cases from Fayoum Governorate in Egypt from March 2014 to December 2014. Cases in the present study were included in 2 groups; the first group comprised 500 cases randomly selected from inhabitants of Abou Shanab and EL-Khaldia villages of Abshoy district in Fayoum. The second group included 100 cases selected from patients admitted to Fayoum Fever Hospital presenting with symptoms and signs suggestive of malaria.

All cases were subjected to detailed history taking with special concern history of antimalarial drug intake and history of travel to malaria endemic areas. Cases were subjected to thorough clinical examination for signs of malaria infection. Blood samples were collected from all cases to prepare thin and thick blood films, transferred to clean sterile dry tubes containing EDTA, and stored at 2°C–8°C for up to 3 days or at −20°C for longer storage. Blood samples were tested by the malaria (pf/pan) one-step rapid test which is a lateral flow chromatographic immunoassay for the simultaneous detection and differentiation of antigens of* Plasmodium* species in human blood samples or serum samples.

### 2.1. Parasitological Examination

Thin blood films were prepared by placing the edge of the spreader slide in a drop of blood and smearing the blood along the surface. Films were allowed to air-dry and were fixed with absolute methanol. For thick blood films, a blood spot was stirred in a circular motion with the corner of the slide and slides were left to dry without fixation. After drying of blood films, they were stained with diluted Giemsa (1 : 20, vol/vol) for 20 minutes and washed in buffered water [21].

### 2.2. Standardization of Microscopic Examination

To ensure good quality of staining and standardization of blood film examination and reporting, the amount of blood used to make blood films, especially thick films, was kept as constant as possible and the blood was spread evenly over a specified area of the slide (15 × 15 mm for thick films). Each slide was subjected to preliminary screening using low power objectives (×10 and ×40) followed by examination of at least 100 microscopic fields using high power objectives (×100). Slides were examined by 2 microscopic experts and suspicious slides were examined by a third expert.

### 2.3. Immunological Test

Stored blood samples were tested for plasmodial antigens using the commercially available malaria pf/pan one-step rapid test [Abon Biopharm (Hangzhou) Co., Ltd., China] which allows detection of malaria antigen in blood flowing along a membrane containing specific anti-malaria antibodies and enables differentiation of* Plasmodium* species in blood samples. The assay was performed according to manufacturer instructions by dispensing 10 *μ*L of blood sample into the sample well and adding three drops of lysis buffer to the buffer well. After 5 minutes, one full drop of buffer was added to the sample well and the results were read after 15 minutes.

**Figure 1:**
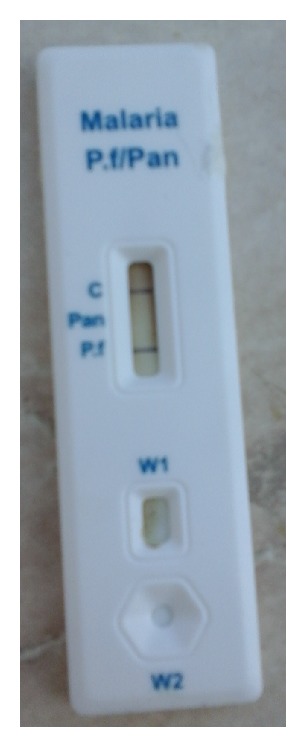
Positive RDT for* Plasmodium falciparum*.

**Figure 2:**
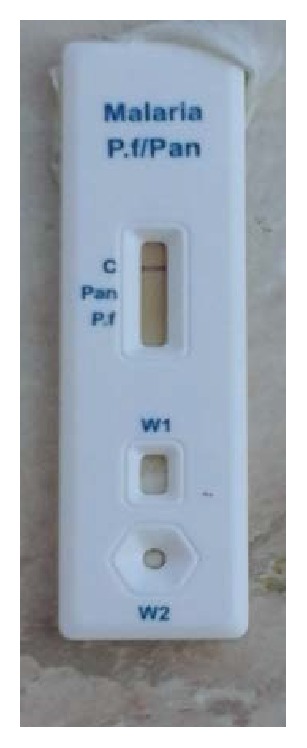
Negative RDT for* Plasmodium falciparum* and* Plasmodium* species.

Presence of C band was an indicator of validity of the test; positive test for* Plasmodium falciparum* infection was indicated by development of pf band in addition to C band (Figure 1) and positive test for* Plasmodium vivax*,* Plasmodium ovale*, or* Plasmodium malariae* was indicated by development of pan band in addition to C band. Negative test for all species was indicated by absence of pf and pan bands in addition to the presence of C band according to the manufacturer's instructions (Figure 2).

### 2.4. Ethical Considerations

Informed written consent was individually signed by each patient before inclusion in the present study. The current study was conducted according to the institutional ethical and professional guidelines in management and follow-up of the cases.

## 3. Results 

A total of 600 cases were included in the present study. Their mean age was 23.7 years (SD: 17.9 years) and 29.3% of cases were males and 70.7% were females. The mean ages of cases in group 1 and group 2 were 23.30 ± 17.7 and 25.89 ± 18.7 years, respectively. All cases were residents of Fayoum Governorate in Egypt and 14.8% of them gave history of travel to El Khartoum in Sudan.

As part of malaria control program in Egypt, 20.5% of cases received antimalarial drugs. The administered drugs included Chloroquine, Coartem® (a combination of artemether and lumefantrine), and Larum® (mefloquine) in 16.7%, 0.3%, and 0.8% of cases, respectively.

Detailed history taking and clinical examination of all cases revealed a variety of clinical manifestations (Table 1) with elevated body temperature (mean 38.6 ± 0.5°C) as the main presenting symptom and pallor as the most frequently detected clinical sign. Other causes of fever and pallor were investigated as such patients were admitted to Fayoum Fever Hospital for further management.

**Figure 3:**
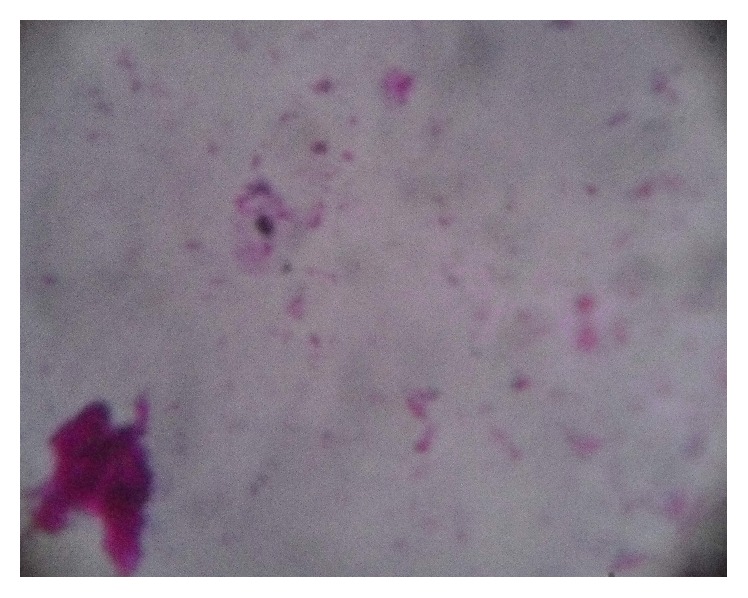
Ring of* Plasmodium falciparum* in thick blood film (×1000).

Microscopic examination of thin and thick blood films of cases included in the first group revealed no plasmodial parasitic stages in all cases. In the second group, microscopic examination of thick blood film revealed ring stage of* Plasmodium falciparum* in one case (Figure 3).

Blood samples of cases were tested for plasmodial antigens using malaria pf/pan one-step rapid test. Three cases in the second group were positive for* P. falciparum* antigen but were negative for other plasmodial antigens. The three positive cases were males with history of travel to El Khartoum in Sudan. They received Coartem for 4 weeks immediately after return from Sudan. Clinically, all of them had fever as the main presenting sign.

## 4. Discussion

Malaria is a major cause of morbidity and mortality that has been identified in Egypt since ancient times. Remnant residual foci are still localized in two districts, Sinnuris and Abshoy, Fayoum Governorate. A wide range of malaria control measures has been implemented including the use of artemisinin combination therapy and improved diagnosis using rapid diagnostic tests (RDTs) [22], aiming to reduce complications and mortality from malaria.

In the present study, a large number of cases presenting with fever or history of fever (64.7%) did not have malaria. Diagnosis based on signs and symptoms has varied sensitivity and specificity depending on clinical features and transmission-associated acquired immunity [23]. In accordance with that, Perkins et al. [24] reported that clinically based diagnosis of malaria showed very low specificity (0–9%) but 100% sensitivity. Similarly, another study showed that use of clinical algorithm for diagnosis of malaria resulted in 30% overdiagnosis of malaria [25]. A large number of patients with fever were screened for malaria of which 26% were positive for* P. falciparum* [26]. In this aspect, it is recommended that clinically suspected malaria should be confirmed parasitologically prior to treatment [27].

Microscopic detection and identification of* Plasmodium* species in Giemsa-stained thick blood films for screening and thin blood films for species' confirmation remain the gold standard for laboratory diagnosis [28]. In our study, thick blood film examination was able to detect the ring stage of* Plasmodium falciparum* in one case. The expected sensitivity that can be achieved by examination of the thick blood film procedure is about 50 parasites/*μ*L of blood which is equivalent to 0.001% of RBC infected [17].

Rapid diagnostic tests for malaria could improve the targeting of antimalarials to true cases of malaria and to distinguish cases of* falciparum* malaria from cases of* vivax* malaria [29].

RDTs for malaria are based on the detection of either histidine-rich protein 2, produced only by* Plasmodium falciparum,* or parasite specific lactate dehydrogenase produced by all four species [30].

In the present study, malaria one-step rapid test pf/pan detected* Plasmodium falciparum* antigen in 3 cases (2 cases of them were missed by microscopy). The highest sensitivity of any RDT for* P. falciparum* was 98% and lowest sensitivity was 76% [26]. For the diagnosis of* P. falciparum* infection, tests detecting PfHRP2 showed high and similar sensitivity (96%). One-step malaria pf/pv detected plasmodial antigen in 2 samples that were missed by aldolase based detection tests [31].

In the present study, there were 2* Plasmodium falciparum* infections missed by microscopy and were detected by RDT probably due to low parasitemia in tested samples or drug intake that clears parasitemia with persistence of antigenemia [32]. Another possible explanation is the sequestration of malaria parasites in deep capillaries leading to decreased number of circulating parasites in peripheral blood [33].

In agreement with our results, Leslie et al. [34] reported that microscopy had a lower operational sensitivity for detection of* P. falciparum* than different rapid diagnostic tests.

Studies comparing the cost of* P. falciparum* diagnosis by RDTs and microscopy found RDTs to be more cost-effective [35]. On the other hand, Yukich [36] found microscopy to be more cost-effective than RDTs. The high cost of the immunochromatographic test for* P. vivax* makes microscopy most cost-effective [37].

In the present study, fever was a constant symptom in malaria positive cases and this was in accordance with the universal screening symptom for malaria in research studies [38]. All positive cases by RDT received Coartem as antimalarial drug and had history of travel to Sudan (El Khartoum). Sudan has one of the highest malaria burdens in Sub-Saharan Africa [39]. The disease is endemic countrywide putting the entire population at risk of infection. Malaria endemicity varies from hypoendemicity, through mesoendemicity and hyperendemicity, to holoendemicity. Parasite prevalence ranges from less than 1% to more than 40% with great variability across the states and is higher in rural areas than in urban areas [40]. Species differentiation in positive samples showed a prevalence of* Plasmodium falciparum* which coincides with high level of* Plasmodium falciparum* activity in Sudan [41].

In conclusion, RDTs were found simple and effective for rapid diagnosis of malaria which might enforce the control measures in Egypt against imported malaria that represents a potential risk of reintroduction of malaria in Egypt.

## Figures and Tables

**Table 1:** Clinical manifestations detected in the study groups.

Clinical manifestations	First group (*n* = 500)		Second group (*n* = 100)	
	Frequency	Percentage	Frequency	Percentage
Pallor	329	65.8%	80	80%
Hepatomegaly	33	6.6%	11	11%
Splenomegaly	43	8.6%	12	12%
Fever	416	83.2%	99	99%
Rigors	80	16%	19	19%
Dark urine	18	3.6%	4	4%
